# Supplementary material for: Developmental plasticity enables an intestinal tapeworm to adapt to dietary stress
Source: Nat Commun. 2026 Feb 20;17:2985. doi: 10.1038/s41467-026-69475-0 (PMC13036029; doi:10.1038/s41467-026-69475-0)

Supplementary Fig. 1 | Cluster-based analysis of diet-dependent gene expression patterns in *Hymenolepis diminuta*.

A, Heatmap showing normalized gene expression across individual biological samples from tapeworms recovered from rats fed a Western diet (WD1–WD3) or an Accessible Fiber diet (AD1–AD3), with three biological replicates per diet ($n = 3$ *per* group). Columns represent individual genes and rows represent individual samples. Gene expression values were scaled by gene (z-score) prior to clustering. Genes were grouped into four expression clusters (Clusters A–D) using k-means clustering based on similarity of expression profiles across dietary conditions. The number of genes assigned to each cluster is indicated below the heatmap. **B,** Mean expression profiles of genes within each cluster across individual samples, illustrating distinct diet-associated transcriptional trajectories. For each cluster, functional enrichment analysis was performed independently. Enrichment of Orthologous Groups (COGs), KEGG pathways, and Pfam domains was assessed using clusterProfiler. The most significantly enriched functional categories for each cluster are shown, summarizing dominant biological processes, pathways and protein domains associated with diet-responsive gene expression programs. Each biological replicate corresponds to *H. diminuta* recovered from a different rat.

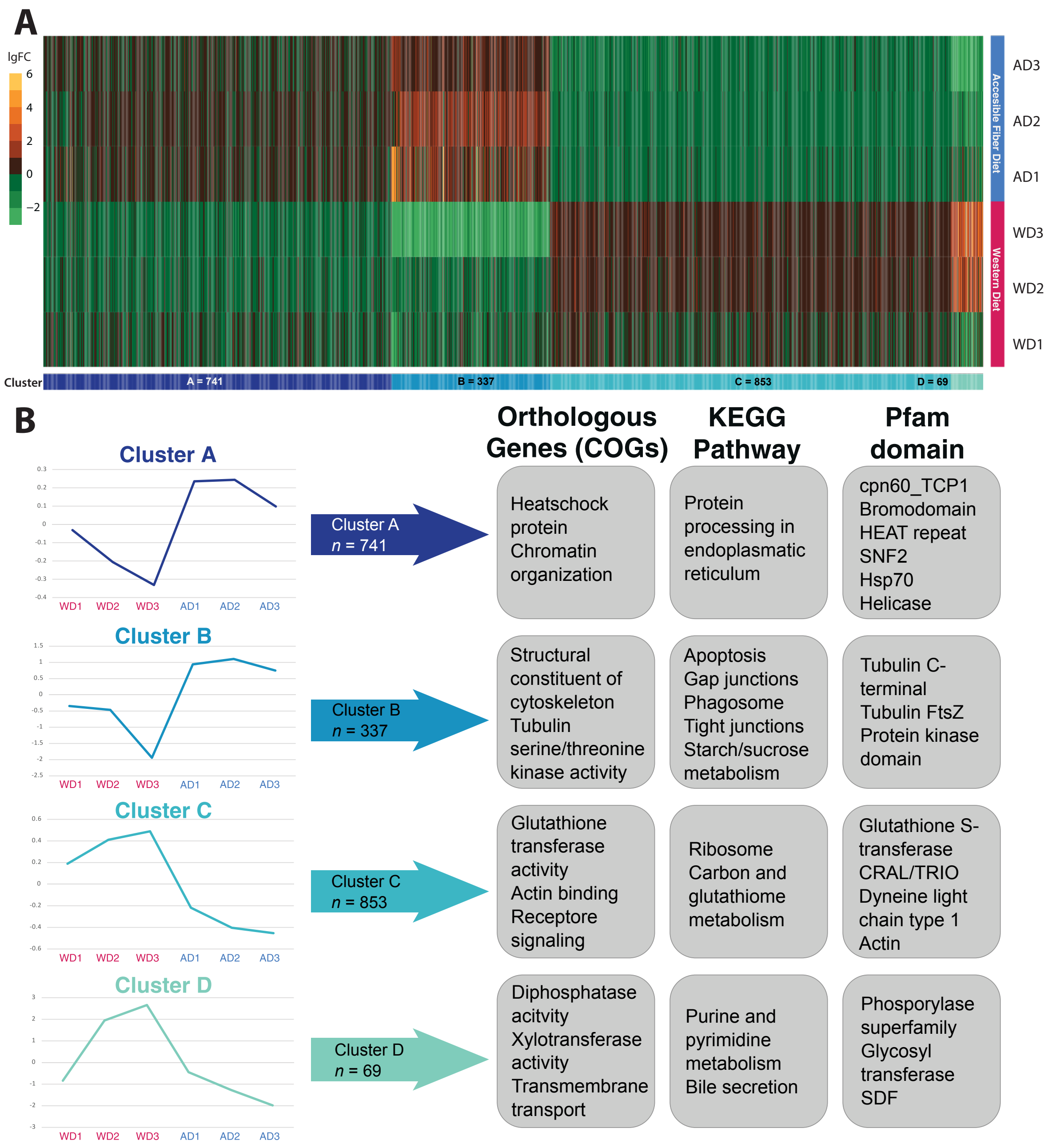

Supplementary Fig. 2 | Differential gene expression analysis of *Hymenolepis diminuta* in response to dietary fiber availability.

Differential gene expression analysis was performed using DESeq2, comparing worms recovered from rats fed a Western diet and an Accessible Fiber diet, with three biological replicates per diet ($n = 3$ *per* group; each replicate corresponds to *H. diminuta* recovered from a different rat). Genes were considered differentially expressed at a false discovery rate (FDR) < 0.01 and an absolute $\log_2$ fold change > 1. **A,** Volcano plot showing the distribution of differentially expressed genes between dietary conditions. Each point represents one gene; genes significantly upregulated under the Western diet are shown in orange, and genes significantly downregulated under the Western diet are shown in green. **B,** Scatter plot comparing normalized gene expression levels between Western diet and Accessible Fiber diet samples, illustrating the magnitude and direction of diet-associated transcriptional changes across all genes. **C,** Bar plot summarizing the total number of differentially expressed genes (DEGs), separated into genes upregulated or downregulated under the Western diet. Exact adjusted P values for all genes are provided in Supplementary Data 1.

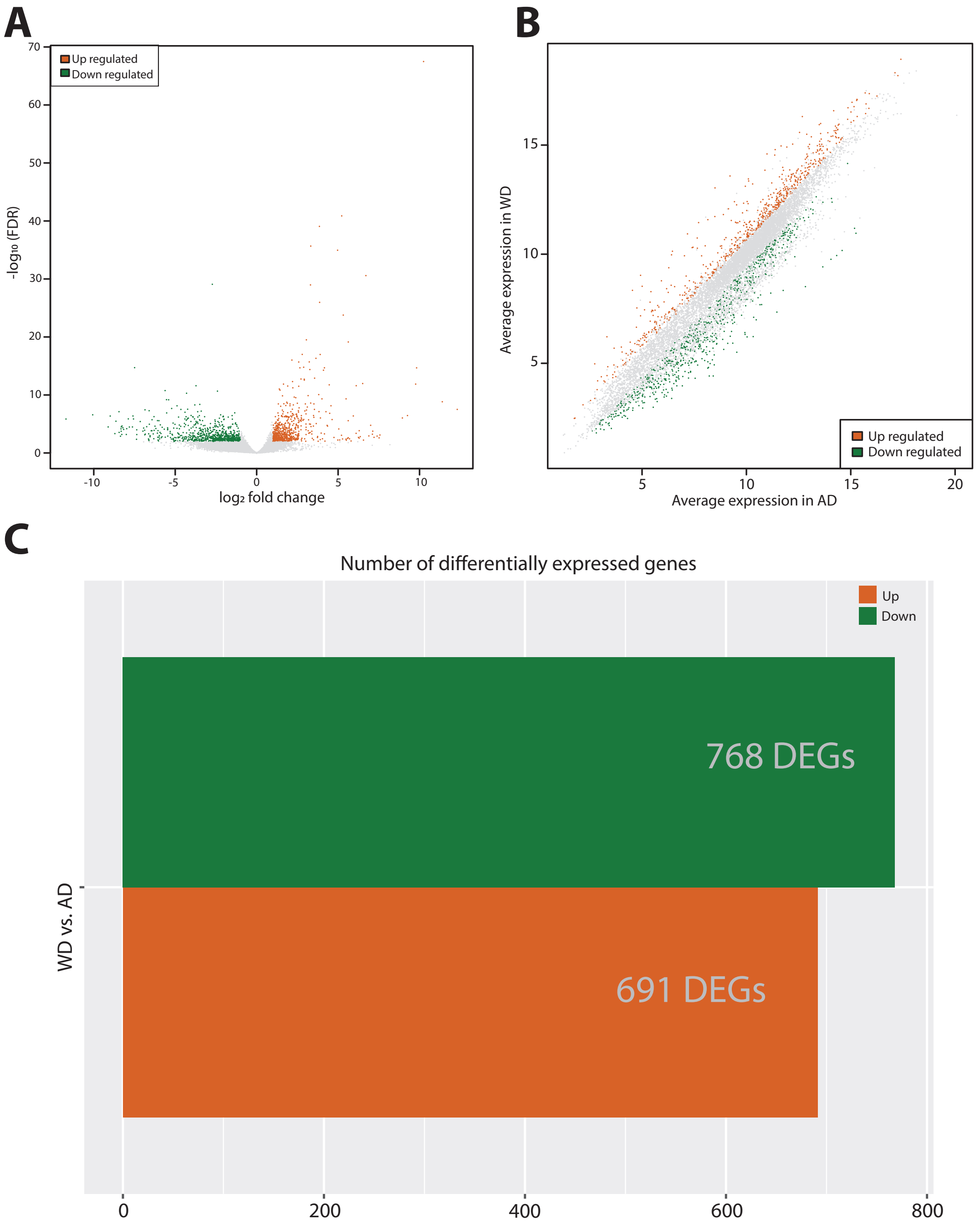

Supplementary Fig. 3 | Pre-processing and exploratory quality assessment of RNA-seq data from *Hymenolepis diminuta*. A, Total read counts per RNA-seq sample, shown in millions. **B,** Boxplots of normalized and transformed gene expression values across samples after normalization, illustrating overall comparability between samples. Boxes represent the interquartile range (25th–75th percentiles), center lines indicate medians, and whiskers extend to the minimum and maximum values. **C,** Density plot showing the distribution of transformed expression values across samples, highlighting consistent global expression profiles across dietary conditions. **D,** Scatter plot comparing transformed expression values between two representative samples (WD1 and WD2), illustrating high concordance between samples. Similar concordance was observed across all pairwise sample comparisons. RNA-seq data were generated from independent biological samples (WD1–WD3 and AD1–AD3), with each sample derived from *H. diminuta* recovered from a different rat. Panels are intended for exploratory quality assessment; no statistical hypothesis testing was performed.

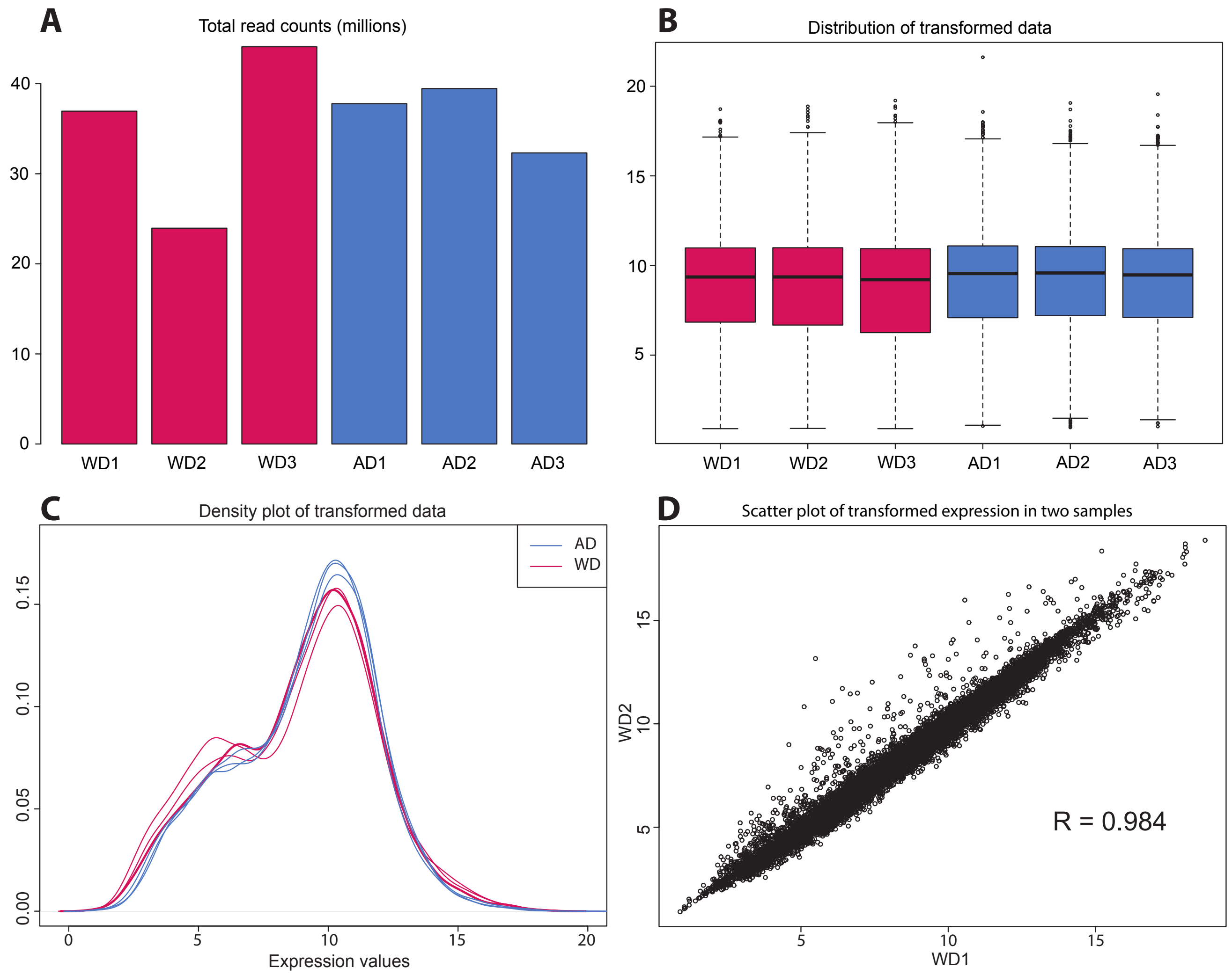

Supplementary Fig. 4 | KEGG pathway visualization of diet-associated differential gene expression in the WNT signaling pathway in *Hymenolepis diminuta*. Differentially expressed genes identified between worms recovered from rats fed a Western diet (WD) and an Accessible Fiber diet (AD) were mapped onto the KEGG WNT signaling pathway using Pathview. Gene expression changes were derived from DESeq2 analysis performed on independent biological samples (WD1–WD3 and AD1–AD3), with each sample corresponding to *H. diminuta* recovered from a different rat. Node colors represent $\log_2$ fold changes in gene expression (WD vs. AD), with orange indicating genes upregulated under the Western diet and green indicating genes downregulated under the Western diet. Asterisks denote genes that were significantly differentially expressed based on DESeq2 analysis (false discovery rate < 0.01; $|\log_2$ fold change$| > 1$). This pathway map represents a visualization of differential expression results and does not constitute an independent statistical analysis.

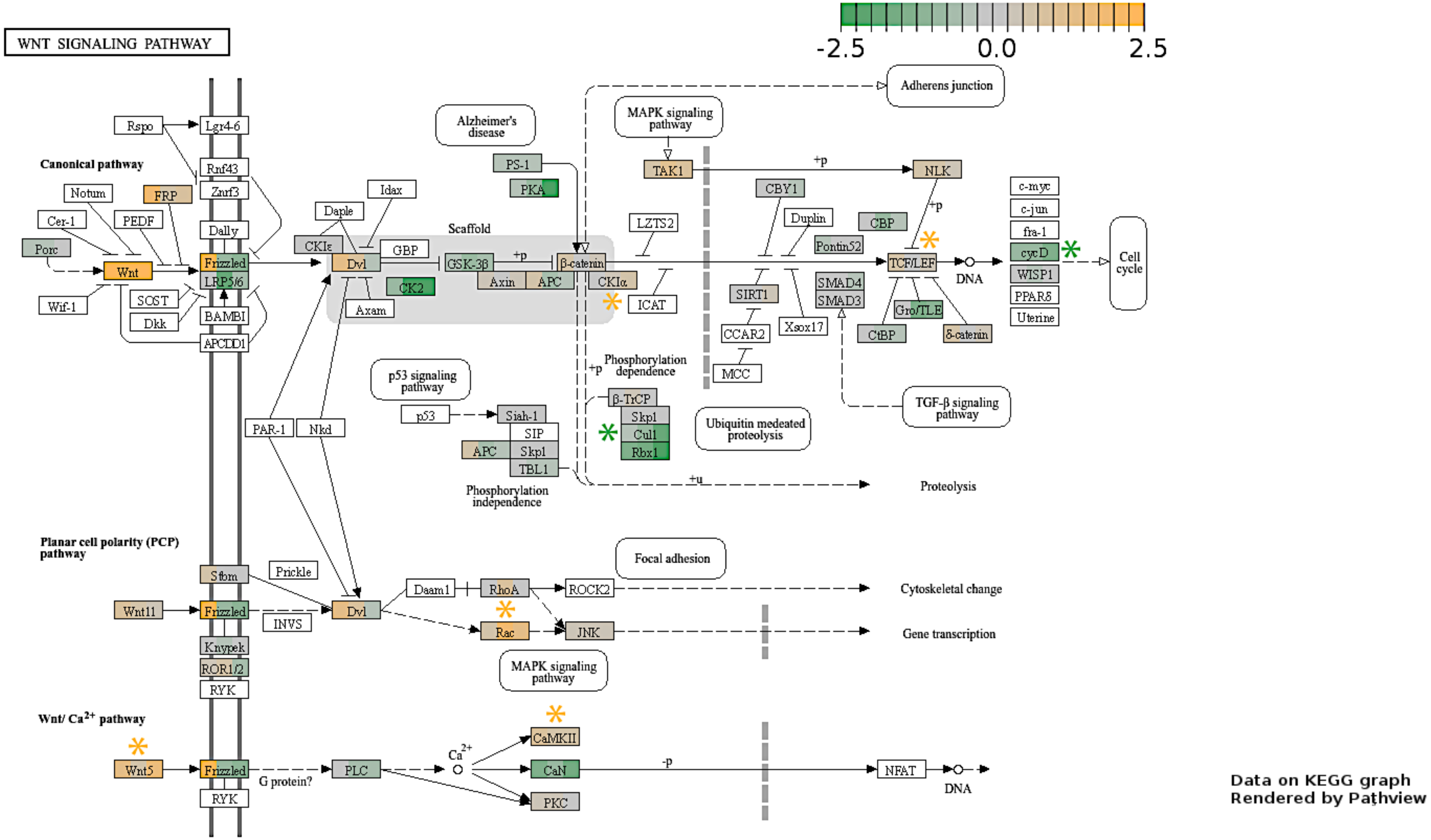

Supplementary Fig. 5 | Body weight dynamics in colonized and non-colonized rats under different dietary conditions.

Body weight was monitored longitudinally in rats fed either an Accessible Fiber diet or a Western diet, with or without *Hymenolepis diminuta* colonization (n = 8 *per* group). Body weight is expressed as percentage change relative to day 0, corresponding to the initiation of experimental diets. **A,** Body weight trajectories across all experimental groups. **B,** Body weight changes in rats fed the Accessible Fiber diet, comparing colonized (A-HD) and non-colonized (A-NC) rats. **C,** Body weight changes in rats fed the Western diet, comparing colonized (W-HD) and non-colonized (W-NC) rats. Data are presented as mean ± SEM. Longitudinal body-weight data were analyzed using three-way repeated-measures ANOVA with time, diet, and colonization as factors, applying the Geisser–Greenhouse correction. Post hoc comparisons between groups at individual time points were performed using Tukey's multiple comparisons test. Statistical significance for post hoc comparisons is indicated as *p = 0.05–0.01, **p = 0.01–0.001, ***p < 0.001).

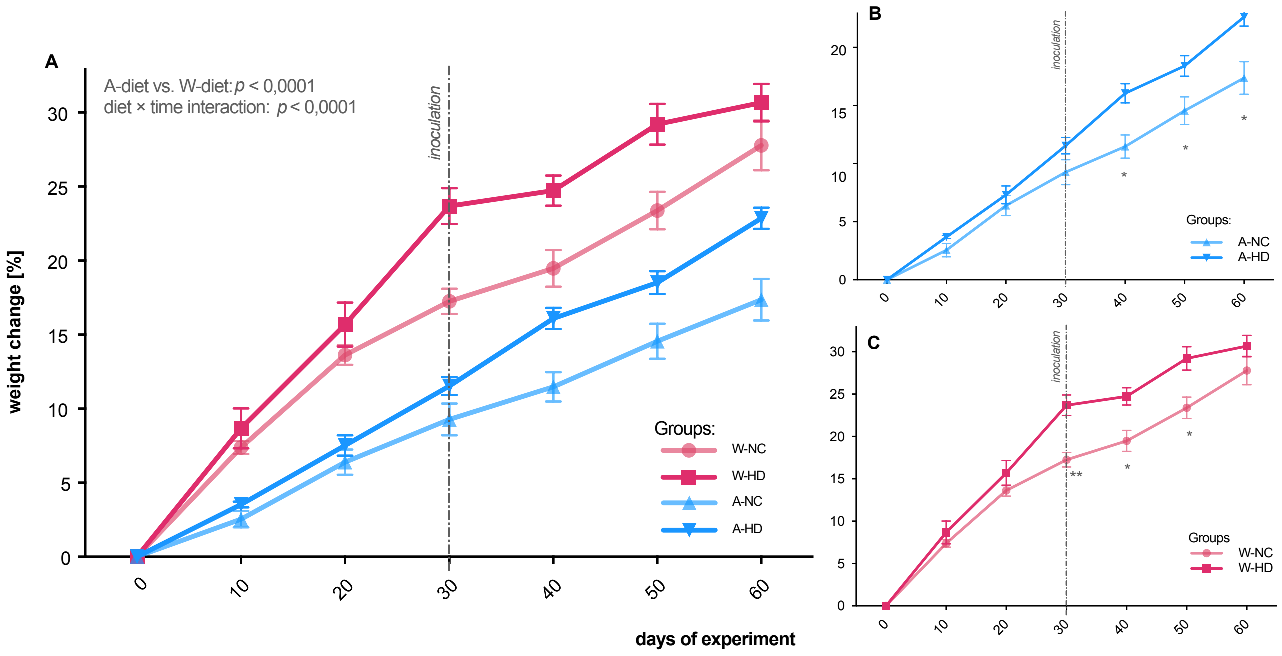

Supplement: Supplementary file 1 — Supplementary Information [file 41467_2026_69475_MOESM1_ESM.pdf]
