# Supplementary material for: Developmental plasticity enables an intestinal tapeworm to adapt to dietary stress
Source: Nat Commun. 2026 Feb 20;17:2985. doi: 10.1038/s41467-026-69475-0 (PMC13036029; doi:10.1038/s41467-026-69475-0)
Supplement: Supplementary file 2 — Description of Additional Supplementary Files [file 41467_2026_69475_MOESM2_ESM.pdf]

Supplementary Data 1 | Differentially expressed genes in adult Hymenolepis diminuta under fiber-depleted dietary conditions. This file contains the list of genes identified as differentially expressed by DESeq2 analysis in adult *H. diminuta* recovered from rats fed a Western (low-fiber) diet compared with an Accessible Fiber diet. Each row represents one gene. Columns report gene identifier, functional annotation (where available), direction of regulation relative to the Accessible Fiber diet (upregulated or downregulated under the Western diet), $\log_2$ fold change ($\log_2FC$), and false discovery rate (FDR). Genes were considered differentially expressed if they met the thresholds $|\log_2FC| \geq 1$ and FDR < 0.01. Functional categories were assigned based on Gene Ontology annotations and curated literature and include developmental signaling pathways, reproduction and cell cycle regulation, cytoskeletal and muscle-associated genes, metabolic pathways, transmembrane transport, cell–cell junction components, stress response, and detoxification.

The data are provided as a separate Excel file.

Supplementary Data 2 | Intestinal content mass and extraction volumes used for metabolomic analysis. This file contains the exact wet mass of small intestinal contents collected from individual rats and the corresponding volumes of extraction solvent used for metabolomic analysis. Each row represents one biological replicate (individual rat). Intestinal contents were dissected, weighed, and extracted in methanol:acetonitrile (MeOH:ACN, v/v) containing 0.1% formic acid using a 1:4 (w/w) ratio of intestinal content to extraction solvent. The data provide source measurements used for normalization and downstream metabolomic analyses.

The data are provided as a separate Excel file.
