# Supplementary material for: Developmental plasticity enables an intestinal tapeworm to adapt to dietary stress
Source: Nat Commun. 2026 Feb 20;17:2985. doi: 10.1038/s41467-026-69475-0 (PMC13036029; doi:10.1038/s41467-026-69475-0)
Supplement: Supplementary file 5 — Reporting Summary [file 41467_2026_69475_MOESM5_ESM.pdf]

nature portfolio

Corresponding author(s): Kateřina Jirků

Last updated by author(s): Jan 30, 2026

Reporting Summary

Nature Portfolio wishes to improve the reproducibility of the work that we publish. This form provides structure for consistency and transparency in reporting. For further information on Nature Portfolio policies, see our Editorial Policies and the Editorial Policy Checklist.

Statistics

For all statistical analyses, confirm that the following items are present in the figure legend, table legend, main text, or Methods section.

n/a	Confirmed	
☐	☒	The exact sample size (*n*) for each experimental group/condition, given as a discrete number and unit of measurement
☐	☒	A statement on whether measurements were taken from distinct samples or whether the same sample was measured repeatedly
☐	☒	The statistical test(s) used AND whether they are one- or two-sided<br>*Only common tests should be described solely by name; describe more complex techniques in the Methods section.*
☐	☒	A description of all covariates tested
☐	☒	A description of any assumptions or corrections, such as tests of normality and adjustment for multiple comparisons
☐	☒	A full description of the statistical parameters including central tendency (e.g. means) or other basic estimates (e.g. regression coefficient) AND variation (e.g. standard deviation) or associated estimates of uncertainty (e.g. confidence intervals)
☐	☒	For null hypothesis testing, the test statistic (e.g. *F*, *t*, *r*) with confidence intervals, effect sizes, degrees of freedom and *P* value noted<br>*Give P values as exact values whenever suitable.*
☐	☒	For Bayesian analysis, information on the choice of priors and Markov chain Monte Carlo settings
☐	☒	For hierarchical and complex designs, identification of the appropriate level for tests and full reporting of outcomes
☐	☒	Estimates of effect sizes (e.g. Cohen's *d*, Pearson's *r*), indicating how they were calculated

Our web collection on statistics for biologists contains articles on many of the points above.

Software and code

Policy information about availability of computer code

Data collection

No software used for data collection

Data analysis

Only established, publicly available software was used; no custom code was generated.

Data collection
RNA sequencing data were generated using the Illumina NextSeq 550Dx platform. Bacterial 16S rRNA gene sequencing was performed on the Illumina MiSeq platform using the MiSeq Reagent Kit v3. Metabolomics data were acquired using an Orbitrap Q Exactive Plus mass spectrometer coupled to a Dionex Ultimate 3000 UHPLC system and Dionex open autosampler (Thermo Fisher Scientific), with data acquisition and primary processing performed using Xcalibur software v2.1. Quantitative PCR data were acquired using a LightCycler 480 system (Roche).

Data analysis and visualization
Statistical analyses and data processing were conducted in R software (versions 3.6–4.3.1; R Core Team), with additional data handling and visualization performed in RStudio v0.99.489 and GraphPad Prism v10. General statistical analyses were implemented using the base stats package in R.

RNA-seq analysis
RNA-seq reads were aligned to the Hymenolepis diminuta genome using STAR v2.7.3a, followed by filtering with SAMtools v1.11. Gene-level read quantification was performed using featureCounts from the subread package v2.0.1. Exploratory transcriptomic analyses were conducted using the IDEP online platform v0.96, including data filtering, transformation, and clustering. Differential gene expression analysis

was performed using DESeq2 v1.38.35. Functional enrichment analyses were conducted using clusterProfiler v4.6.2 and topGO v2.50.0. Data visualization was performed using the tidyverse v1.3.2, viridis v0.6.4, and pheatmap v1.0.12 R packages.

Microbiome analysis
Primer trimming was performed using skewer v0.2.2. Amplicon sequence variant inference and denoising were carried out using DADA2 v1.18. Taxonomic assignment was performed using the naïve Bayesian RDP classifier trained on the SILVA v138 reference database. Microbiome data were analyzed in R using the vegan and phyloseq packages for diversity analyses, community dissimilarity testing, and principal coordinates analysis. Microbiome biomarker discovery was performed using the LEfSe method.

Metabolomics data analysis
Metabolomics data processing and metabolite annotation were performed using the in-house software MetaboliteMapper in combination with Xcalibur v2.1. Statistical evaluation of metabolomics data was conducted using CANOCO v4.5, applying detrended correspondence analysis, principal component analysis, redundancy analysis, and Monte Carlo permutation tests.

Quantitative PCR and immune marker analysis
Relative gene expression from qPCR data was calculated using the Pfaffl method implemented in R software v4.3.1. Statistical group comparisons were performed using Kruskal–Wallis tests followed by Dunn's multiple comparisons tests. Data visualization was performed using GraphPad Prism v10.

For manuscripts utilizing custom algorithms or software that are central to the research but not yet described in published literature, software must be made available to editors and reviewers. We strongly encourage code deposition in a community repository (e.g. GitHub). See the Nature Portfolio guidelines for submitting code & software for further information.

Data

Policy information about availability of data

All manuscripts must include a data availability statement. This statement should provide the following information, where applicable:

- Accession codes, unique identifiers, or web links for publicly available datasets
- A description of any restrictions on data availability
- For clinical datasets or third party data, please ensure that the statement adheres to our policy

Data Availability
Transcriptomic data generated in this study are deposited in the NCBI Sequence Read Archive under accession code PRJNA1126432, including raw sequencing reads SRR29488123–SRR29488126 and SRR29488128. Bacteriome sequencing data are archived in the European Nucleotide Archive under accession code PRJEB86956. Metabolomics data and additional source data supporting the findings of this study—including images and videos of Hymenolepis diminuta, worm length measurements, egg counts, cytokine relative and Cp values, raw and relative body weight data, a checklist of bacteriome sequences, and a table of metabolite annotations and standards are available in the Figshare repository under accession DOI: XXXX [https://figshare.com/s/df77505840944a777748]. Detailed methodological protocols are available from the corresponding author upon request.

Research involving human participants, their data, or biological material

Policy information about studies with human participants or human data. See also policy information about sex, gender (identity/presentation), and sexual orientation and race, ethnicity and racism.

Reporting on sex and gender	*Use the terms sex (biological attribute) and gender (shaped by social and cultural circumstances) carefully in order to avoid confusing both terms. Indicate if findings apply to only one sex or gender; describe whether sex and gender were considered in study design; whether sex and/or gender was determined based on self-reporting or assigned and methods used. Provide in the source data disaggregated sex and gender data, where this information has been collected, and if consent has been obtained for sharing of individual-level data; provide overall numbers in this Reporting Summary. Please state if this information has not been collected. Report sex- and gender-based analyses where performed, justify reasons for lack of sex- and gender-based analysis.*
Reporting on race, ethnicity, or other socially relevant groupings	*Please specify the socially constructed or socially relevant categorization variable(s) used in your manuscript and explain why they were used. Please note that such variables should not be used as proxies for other socially constructed/relevant variables (for example, race or ethnicity should not be used as a proxy for socioeconomic status). Provide clear definitions of the relevant terms used, how they were provided (by the participants/respondents, the researchers, or third parties), and the method(s) used to classify people into the different categories (e.g. self-report, census or administrative data, social media data, etc.) Please provide details about how you controlled for confounding variables in your analyses.*
Population characteristics	*Describe the covariate-relevant population characteristics of the human research participants (e.g. age, genotypic information, past and current diagnosis and treatment categories). If you filled out the behavioural & social sciences study design questions and have nothing to add here, write "See above."*
Recruitment	*Describe how participants were recruited. Outline any potential self-selection bias or other biases that may be present and how these are likely to impact results.*
Ethics oversight	*Identify the organization(s) that approved the study protocol.*

Note that full information on the approval of the study protocol must also be provided in the manuscript.

Field-specific reporting

Please select the one below that is the best fit for your research. If you are not sure, read the appropriate sections before making your selection.

- [x] Life sciences
- [ ] Behavioural & social sciences
- [ ] Ecological, evolutionary & environmental sciences

For a reference copy of the document with all sections, see nature.com/documents/nr-reporting-summary-flat.pdf

Life sciences study design

All studies must disclose on these points even when the disclosure is negative.

Sample size	Sample sizes were determined based on the biology of Hymenolepis diminuta, prior pilot observations, and the specific analytical requirements of each experiment, while adhering to the 3R principle (Replacement, Reduction, Refinement). In Experiment 1, a total of 88 rats were used and assigned to experimental groups based on diet (Western diet or Accessible Fiber diet) and colonization status. Group sizes were informed by pilot observations indicating arrested or reduced development of H. diminuta under the Western diet and an approximately 50% colonization success rate, which necessitated increased numbers of rats in colonized Western diet groups. Colonized animals were further allocated to mutually exclusive analytical branches (worm-centered vs. host-centered analyses) due to incompatibility of intact worm recovery with simultaneous intestinal content sampling. In Experiment 2, 18 rats (n = 6 per group) were used to assess reversibility of diet-induced changes in adult tapeworm condition. Sample size was informed by a pilot experiment monitoring egg production dynamics, indicating that this number was sufficient to detect biologically meaningful differences. Overall, the chosen sample sizes were sufficient to capture biologically relevant effects and ensure reproducibility, while remaining compliant with ethical standards and the 3R principle.
Data exclusions	Animals assigned to colonized groups but lacking confirmed presence of Hymenolepis diminuta at necropsy were excluded from analyses requiring confirmed colonization status. No other data were excluded.
Replication	For each experiment, a pilot study was first conducted using a smaller number of animals to characterize the biological response of the system and to optimize the experimental design. In the case of Experiment 1, the pilot study revealed reduced tapeworm growth and an approximately 50% colonization success rate under the Western diet, which was taken into account in the design of the main experiment, group size allocation, and assignment of animals to the respective analytical branches. Based on the outcomes of Experiment 1, Experiment 2 was subsequently designed to test the reversibility of diet-induced changes. Here, a pilot study was likewise used to define the experimental design, timing, and outcome measures.
Randomization	Rats were randomly assigned to dietary and colonization groups to ensure balanced representation and comparability between conditions. In Experiment 1, randomization was stratified by diet and colonization. In Experiment 2, randomization was applied to dietary transitions testing reversibility of estivation. All animals were housed under identical conditions to minimize confounders.
Blinding	Blinding was not applied, as it was not feasible for this experimental design. However, outcome measures (egg counts, molecular readouts, sequencing) were objective, and standardized protocols were followed to minimize potential bias and ensure reproducibility.

Reporting for specific materials, systems and methods

We require information from authors about some types of materials, experimental systems and methods used in many studies. Here, indicate whether each material, system or method listed is relevant to your study. If you are not sure if a list item applies to your research, read the appropriate section before selecting a response.

Materials & experimental systems

n/a	Involved in the study	
x		Antibodies
x		Eukaryotic cell lines
x		Palaeontology and archaeology
	x	Animals and other organisms
x		Clinical data
x		Dual use research of concern
x		Plants

Methods

n/a	Involved in the study	
x		ChIP-seq
x		Flow cytometry
x		MRI-based neuroimaging

Animals and other research organisms

Policy information about studies involving animals; ARRIVE guidelines recommended for reporting animal research, and Sex and Gender in Research

Laboratory animals	Laboratory animals<br>Outbred female Wistar rats (RccHan®:WIST; Rattus norvegicus), 13 weeks of age, were obtained from Envigo RMS B.V. (Horst, the Netherlands; the supplier Anlab s.r.o., Prague, Czech Republic), acclimated for seven days prior to the experiment, housed under controlled laboratory conditions with ad libitum access to food and water, and euthanized under anesthesia at the end of the study.

Wild animals	not applicable
Reporting on sex	Only female rats were used; sex differences were not assessed.
Field-collected samples	not applicable
Ethics oversight	This study was approved by the Committee on the Ethics of Animal Experiments of the Biology Centre of the Czech Academy of Sciences (České Budějovice, Czechia, permit no. 33/2018) and by the Resort Committee of the Czech Academy of Sciences (Prague, Czech Republic) according in strict accordance with Czech legislation (Act No. 166/1999 Coll. on Veterinary Care and on Changes of Some Related Laws, and Act No. 246/1992 Coll. on the Protection of Animals against Cruelty), as well as the legislation of the European Union.

Note that full information on the approval of the study protocol must also be provided in the manuscript.

Plants

Seed stocks	*Report on the source of all seed stocks or other plant material used. If applicable, state the seed stock centre and catalogue number. If plant specimens were collected from the field, describe the collection location, date and sampling procedures.*
Novel plant genotypes	*Describe the methods by which all novel plant genotypes were produced. This includes those generated by transgenic approaches, gene editing, chemical/radiation-based mutagenesis and hybridization. For transgenic lines, describe the transformation method, the number of independent lines analyzed and the generation upon which experiments were performed. For gene-edited lines, describe the editor used, the endogenous sequence targeted for editing, the targeting guide RNA sequence (if applicable) and how the editor was applied.*
Authentication	*Describe any authentication procedures for each seed stock used or novel genotype generated. Describe any experiments used to assess the effect of a mutation and, where applicable, how potential secondary effects (e.g. second site T-DNA insertions, mosiacism, off-target gene editing) were examined.*
